# Developmental profiles of child behavior problems from 18 months to 8 years: The protective effects of structured parenting vary by genetic risk

**DOI:** 10.1017/S0954579422000839

**Published:** 2022-08-05

**Authors:** Leslie D. Leve, Daniel Anderson, Gordon T. Harold, Jenae M. Neiderhiser, Misaki N. Natsuaki, Daniel S. Shaw, Jody M. Ganiban, David Reiss

**Affiliations:** 1 Prevention Science Institute, University of Oregon, Eugene, OR, USA; 2 Behavioral Research and Teaching, University of Oregon, Eugene, OR, USA; 3 Always Be Learning, Inc, San Francisco, CA, USA; 4 Faculty of Education, University of Cambridge, Cambridge, UK; 5 Department of Psychology, The Pennsylvania State University, University Park, PA, USA; 6 Department of Psychology, University of California, Riverside, Riverside, CA, USA; 7 Department of Psychology, University of Pittsburgh, Pittsburgh, PA, USA; 8 Department of Psychological and Brain Sciences, George Washington University, Washington, DC, USA; 9 Child Study Center, Yale University, New Haven, CT, USA

**Keywords:** adoption, behavior problems, childhood, genetic, parenting

## Abstract

Some children are more affected by specific family environments than others, as a function of differences in their genetic make-up. However, longitudinal studies of genetic moderation of parenting effects during early childhood have not been conducted. We examined developmental profiles of child behavior problems between 18 months and age 8 in a longitudinal parent–offspring sample of 361 adopted children. In toddlerhood (18 months), observed structured parenting indexed parental guidance in service of task goals. Biological parent psychopathology served as an index of genetic influences on children’s behavior problems. Four profiles of child behavior problems were identified: low stable (11%), average stable (50%), higher stable (29%), and high increasing (11%). A multinominal logistic regression analysis indicated a genetically moderated effect of structured parenting, such that for children whose biological mother had higher psychopathology, the odds of the child being in the low stable group increased as structured parenting increased. Conversely, for children whose biological mother had lower psychopathology, the odds of being in the low stable group was reduced when structured parenting increased. Results suggest that increasing structured parenting is an effective strategy for children at higher genetic risk for psychopathology, but may be detrimental for those at lower genetic risk.

It is widely recognized that children with elevated behavioral and emotional problems during early childhood are more likely than those with lower levels of these problem behaviors to experience academic, social, and emotional challenges in adolescence and beyond (Brennan et al., 2012; Broidy et al., 2003; Caspi, Moffitt, Newman, et al., 1996; Davis et al., 2015; Pingault et al., 2011; Reef et al., 2011). Two dimensions of behavioral and emotional problems are typically studied: externalizing problems (e.g., aggression, rule-breaking, and delinquency) and internalizing problems (e.g., anxious, withdrawn, and somatic complaints). The field of developmental psychopathology has often focused on total behavior problems based on the co-occurrence of externalizing and internalizing behaviors during childhood (e.g., Oh et al., 2020; Wadsworth et al., 2001). The purpose of this study is to advance the understanding of the familial transmission of psychopathology by examining the protective and negative effects of parenting in the context of higher and lower genetic risk.

We use data from a longitudinal parent–offspring adoption study to examine whether an observed measure of structured parenting during toddlerhood that taps aspects of parental structure and control would predict profiles of total behavior problems from 18 months to age 8. The adoption design allows for an examination of environmental transmission of risk from nonbiological parents. It also permits an examination of whether associations between parenting and children’s behavior problem profiles are similar for children with higher versus lower genetic risk for behavior problems by using the biological parents’ psychopathology scores to measure the genetic transmission of risk. By comparison, in studies of genetically related parents and children, genetic, and environmental influences are fully confounded because children are reared by their biological parents. The current work extends a prior study with this same adoption sample that identified a novel genetically moderated cross-sectional association between structured parenting and child behavior problems during toddlerhood (Leve et al., 2009). Using the same measure of toddler parent–child interaction and the same biological parent measures of psychopathology, we now examine the prediction of longitudinal profiles of child behavior problems to age 8. To our knowledge, this study is the first examination of whether a genetically moderated parenting effect on behavior problems during toddlerhood is sustained over time, in this case to age 8.

## Developmental patterns of behavior problems beginning in toddlerhood

There is a large body of research examining changes in children’s internalizing and externalizing behavior problems from toddlerhood to later childhood (Bub et al., 2007; Gilliom, & Shaw, 2004; Liu, 2019; Mesman et al., 2009; Miner & Clarke-Stewart, 2008; Owens & Shaw, 2003; Prinzie et al., 2005; Wiggins et al., 2015; Williams et al., 2009). This research generally suggests a developmental pattern of reductions in both externalizing and internalizing behavior from toddlerhood to formal school entry across both clinical and nonclinical samples. This developmental pattern aligns with what we know about development across childhood: autonomy struggles during the “terrible twos”, followed by an increase in self-regulation, cognitive skills, and social skills in the preschool and school entry periods that lead to fewer expressed behavior problems as children become cognitively and behaviorally better equipped to successfully regulate their behavior (Scaramella & Leve, 2004; Shaw & Bell, 1993).

In addition to developmental patterns over time, prior studies also provide evidence for between-individual differences in change over this developmental period, with some children showing stable behavior problems or increasing behavior problems from toddlerhood to later childhood, whereas the majority of children show a decline in problems. For example, Bub et al. (2007) used data from the National Institute of Child Health and Human Development Study of Early Child Care and Youth Development to examine internalizing and externalizing behavior from age 24 months to the first grade across five time points. On average, children’s internalizing and externalizing behavior declined between 24 months and formal school entry (i.e., first grade); however, there was evidence of between-individual variation in change, with some children showing increases in behavior problems across this developmental period. This suggests that there are groups of children who show more behavior problems over time and other groups who show stable or decreasing problems. Person-centered analysis approaches can help identify such groupings and their predictors, ultimately allowing for a richer understanding of mechanisms of familial transmission of psychopathology.

There is also strong evidence that externalizing and internalizing behaviors co-occur during toddlerhood and later childhood (Basten et al., 2016; Oh et al., 2020; Wadsworth et al., 2001; Wiggins et al., 2015). The high co-occurrence between externalizing and internalizing problems led to the decision to examine total behavior problems in a prior report from the current sample (Leve et al., 2009); an approach which we continue in the current study.

## Prediction of developmental patterns of behavior problems

It is well documented that behavior problems tend to run in families, with the root causes of familial continuity attributed to both environmental factors (e.g., parenting, social context) and genetic factors (e.g., Bartels et al., 2007; Natsuaki et al., 2014; Rhee & Waldman, 2009). Despite a rich body of evidence there is still much we do not understand about the familial transmission of behavior problems in childhood. A systematic review of articles on child internalizing and externalizing behavior measured with the Child Behavior Checklist (CBCL) between child ages 3–6 years old and published from January 2001 to December 2014 (*n* = 28 articles) identified three main groups of risk and protective factors associated with children’s behavior problems: parental/parenting factors, child factors, and environmental factors (Carneiro et al., 2016). The current study focuses on the first two factors, more specifically, on structured parenting as the parenting factor (observed in the home using a coding system that taps aspects of parental structure and control) and on children’s genetic risk for behavior problems (measured using biological parents’ psychopathology) as the child factor. We also include infant fear and impulsivity in order to control for earlier temperamental qualities that might influence children’s subsequent behavior problems and parenting behaviors.

### Structured parenting during toddlerhood

Specific parenting behaviors during early childhood have routinely been found to predict behavior problems over time (e.g., Gilliom & Shaw, 2004). Furthermore, randomized prevention trials indicate that improvements in parenting often mediate intervention effects on child psychopathology, with such improvements leading to reductions in child disruptive behavior problems (Dishion et al., 2008; Gardner et al., 2007). This body of work suggests a causal role of specific parenting behaviors on risk for behavior problems beginning in early childhood. As described earlier, across early childhood, there is a general trend for declining trajectories of behavior problems (e.g., Mesman et al., 2009; Owens & Shaw, 2003). However, children who experience negative parenting often do not show this decline, and instead show stable or increasing behavior problems (Gilliom & Shaw, 2004; Kingston & Prior, 1995). For example, a systematic review of risk factors for internalizing and externalizing problems between ages 3 and 6 found that disciplinary practices such as harsh discipline were associated with higher behavior problems across this developmental period (Carneiro et al., 2016).

In contrast, structured and guiding parenting can aid the development of children’s regulatory capacities (Denham et al., 2000; Holden & West, 1989; Lecuyer & Houck, 2006; Ravindran et al., 2021). This type of structured parenting includes specific parenting behaviors that provide support, instruction, and limit setting to guide child behavior, such as instructing the child to perform a specific step or task when it is time to clean up their toys, rather than simple reactions to child responses (Denham et al., 2000). Structured parenting has been shown to be more beneficial than simple limit setting and instruction, and to contribute to later social competence and self-regulation in children (Houck & Lecuyer-Maus, 2004; Lecuyer & Houck, 2006; Ravindran et al., 2021). For example, in a rural, economically strained sample, parental structuring (in contrast with parental directives) in response to 18-month-olds’ negative emotions was observed during a home visit and found to predict better self-regulation in children between 18 and 48 months (Ravindran et al., 2021). Structured parenting has also been shown to help prevent the development of emotional and behavioral problems early in life (Denham et al., 2000; Gardner et al., 1999; Holden & West, 1989; Holden, 1983; Natsuaki et al., 2013), with particular benefits in preventing risk for behavior problems when children are in social situations that demand compliance (Denham et al., 2000; Gardner et al., 1999). The benefits of structured parenting on reducing child problems have been shown across multiple contexts, including supermarket trips (Holden, 1983), forbidden toy tasks (Holden & West, 1989; Lecuyer & Houck, 2006), and competitive games (Denham et al., 2000). Structured parenting appears to be particularly relevant during toddlerhood based on children’s developmental needs and skills, their need for assistance in managing their emotions and behaviors that they cannot manage independently, and the higher frequency with which parental structuring and directing occur during toddlerhood (Brownell & Kopp, 2010; Ravindran et al., 2021).

A recent longitudinal study that followed children from ages 3 to 8 years found that structured parenting at age 5 was negatively related to child internalizing and attention-academic problems at age 8 (Liu et al., 2020). Furthermore, as discussed later, children’s temperamental inhibition (i.e., lower levels) predicted higher rates of later structured parenting (Liu et al., 2020). Central to the current study, a prior report using the current sample identified structured parenting at 18 months of age as a predictor of children’s behavior problems cross-sectionally (Leve et al., 2009), but only for children with a genetic predisposition for behavior problems. In that study, a cross-over interaction effect was identified such that structured parenting was protective against behavior problems for toddlers at higher genetic risk but was promotive of behavior problems for toddlers at lower genetic risk. This novel interaction suggested that the impact of structured parenting during toddlerhood may vary depending on children’s genetic make-up. However, the 2009 report was cross-sectional, and it is not clear whether structured parenting in toddlerhood has sustained effects on behavior problem profiles into later childhood and if there are different patterns of results for children with varying levels of genetic liability. The current study sought to address this knowledge gap and examine associations between structured parenting during toddlerhood and developmental profiles of total behavior problems from 18 months to age 8.

### Genetic influences on child behavior problems

Much of the prior work on associations between specific parenting behaviors and developmental profiles of behavior problems has been conducted in families where one or both rearing parents are genetically related to the child. Such approaches fully confound genetic and environmental influences on child behavior problems, because the rearing parents provide both genes and the rearing environment to the child. We know from twin studies that genetic influences are important for the development of both externalizing and internalizing problems in childhood, and that there are changes in the magnitude of genetic and environmental influences on behavior problems across childhood (e.g., Bartels et al., 2007; Carroll et al., 2021; Deater-Deckard, 2000; Gjone & Stevenson, 1997; Hoekstra et al., 2008; van der Valk et al., 2003). Twin studies examining behavior problems from toddlerhood to later childhood have found that continuity in behavior problems is primarily explained by genetic and shared environmental factors (non-genetic influences that account for the similarity in family members), whereas primarily genetic and nonshared environmental factors account for change in behavior problems during this developmental period (Bartels et al., 2004, 2007; van Beijsterveldt et al., 2003; van der Valk et al., 2003).

However, prior longitudinal twin modeling approaches have not considered the interaction of genetic and environmental influences during early childhood that may precipitate a specific developmental trajectory of behavior problems later in development. It is clear that children’s genetic propensities can interact with the quality of parenting to predict behavior problems, with proximal aspects of a child’s social context moderating genetic liability or vice versa (e.g., Shanahan & Hofer, 2005). However, the majority of studies investigating how parenting and genetic influences interact have focused on adolescents and adults. For example, during adolescence, genetic influences were greater for antisocial behavior when parenting was more negative or less warm (Feinberg et al., 2007). From child ages 11–14, Burt et al. (2005) demonstrated with a longitudinal twin study that parent–child conflict partially resulted from parental responses to their child’s heritable externalizing behavior, while simultaneously contributing to child externalizing via environmental mechanisms. In young adulthood, one longitudinal study found that genetic moderation of environmental influences was proximally and developmentally limited, with genetic influences on externalizing being greater in the context of more parent–child relationship problems at age 18, but not present at age 25 (Samek et al., 2015).

These adolescent and young adult findings suggest that gene–environment interactions may be time-limited and, perhaps, confined to adolescence. Yet, few studies have examined if the effects of specific parenting behaviors on behavior problems during toddlerhood are conditioned by genetic influences, and no studies have tested whether gene–environment interactions identified in toddlerhood are time-specific versus sustained across a longer period of development. The adoption design is an ideal method for filling this gap because genetic and environmental influences can be disaggregated by including information from rearing parents as well as biological parents. Evidence from a study of older children (ages 7.5–14) provides the first indication that environmental influences on *trajectories* of behavior problems may be conditioned by genetic influences using polygenic scores. In that study, children with lower levels of family instability and lower polygenic risk exhibited a steeper decline in aggression from ages 7.5–14 (Womack et al., 2021). In an earlier report with the sample used in the current report, we identified that adopted children who were at higher genetic risk (as evidenced by their biological parents’ higher levels of psychopathology) only showed higher behavior problems when their adoptive mothers used lower levels of structured parenting; more structured parenting buffered the potential adverse effects of genetic risk for such children. Conversely, higher levels of structured parenting were associated with more behavior problems among children with lower genetic risk (Leve et al., 2009). However, based on the prior study’s cross-sectional design, it remains to be seen whether this interaction would persist in differentiating patterns of problem behavior from ages 18 months to 8 years.

The primary question in the current investigation is to investigate what happens longitudinally after evidence of genetic moderation of a parenting effect is identified in toddlerhood. To our knowledge, no study has yet been conducted to examine whether genetically moderated parenting effects identified during early childhood have a sustained effect on behavior problems longitudinally. Whether such moderated effects persist over time has clear developmental implications and implications for the potential of genetically informed studies to guide the identification of malleable prevention targets.

### Children’s temperamental fear and impulsivity

Children bring their own characteristics to the parent-child relationship that are associated with later behavior problems (Carneiro et al., 2016) and can alter the way that parents interact with their child (e.g., Bell, 1968; Klahr & Burt, 2014). For example, a systematic review suggested that low inhibitory control and anger were associated with more behavior problems in early childhood (Carneiro et al., 2016), a meta-analysis found that self-regulation in preschool was negatively associated with internalizing and externalizing problems in the early school-age years (age 8) (Robson et al., 2020), and a research review indicated that negative emotionality, behavioral inhibition, and self-regulation during infancy predicted psychopathology in childhood and adolescence (Kostyrka-Allchorne et al., 2020). Emotional fear/distress to novelty and behavioral impulsivity/distress to limitations are among the two most widely studied child temperamental characteristics in early childhood linked to later behavior problems, often in combination with specific forms of parenting (e.g., Degnan et al., 2010; Leve et al., 2005). For example, child impulsivity in infancy was most strongly related to externalizing symptoms in middle childhood when parents used noncontrolling parenting strategies (Bates et al., 1998). A study of children followed across a 1-year period in toddlerhood found that compared to children low in irritable temperament, children high in irritable temperament (e.g., anger, frustration, and fearfulness) exhibited more behavior problems in contexts of high interparental conflict, but also exhibited fewer behavior problems in contexts of low levels of interparental conflict (Hentges et al., 2015), suggesting that some children are more susceptible to family environments than other children as a function of their irritable temperament. Due to the preponderance of evidence that child temperament may influence later behavior problems and may also influence later parenting, we controlled for infant temperamental characteristics (distress to limitations and fearfulness) at 9 months, before our first measure of structured parenting and child behavior problems was collected at 18 months.

## The prospective parent–offspring adoption design

The current study uses a prospective adoption design to investigate familial transmission and potential genetic moderation of parenting effects on developmental profiles of behavior problems from age 18 months to age 8 years. The adoption design can be considered a type of natural experiment to examine familial transmission of psychopathology because the adopted children are raised in families in which they have no genetic relationship with their parents (Haugaard & Hazan, 2003). In contrast to studies of genetically related parents and children, similarities between the rearing parents and child in an adoption study can only be attributed to post-natal environmental mechanisms (when adoption design assumptions are met and/or controlled for, as they were in the current study). Conversely, similarities between the child and the biological parents can be attributable to genetic (or prenatal) origins, and their role in moderating parenting effects can be examined. Within this design, biological parent psychopathology serves as an indicator of genetic influences, and specific parenting behaviors are measured in the adoptive family rearing environment. Earlier reports from this study have identified several such genetically moderated parenting effects in toddlerhood (Leve et al., 2019). For example, in children with biological parents with higher levels of psychopathology, greater adoptive parent internalizing behavior predicted higher levels of 9-month old’s attention to frustrating events, a precursor to externalizing behavior (Leve et al., 2010). Additionally, less responsive adoptive mother behavior predicted greater fussiness at 18 months of age for certain children (Natsuaki et al., 2010). However, whether specific environments continue to interact with genetic risk to predict profiles of behavior problems across multiple developmental periods has yet to be tested.

## Current study

To better understand the familial transmission of behavior problems and to clarify “for whom” parenting matters, we investigated how structured parenting during toddlerhood and genetic influences interact to predict developmental profiles of behavior problems from 18 months to age 8. The inclusion of six waves of child data provides a unique opportunity to examine whether a previously identified genetically moderated effect of structured parenting on child behavior problems at age 18 months (Leve et al., 2009) continues to be valid longitudinally. That is, does this same measure of structured parenting at 18 months predict developmental profiles of behavior problems from age 18 months to age 8, and is the association conditioned by children’s genetic risk for behavior problems?

We used latent profile analysis to identify distinct developmental profiles of children’s behavior problems from age 18 months to age 8 years. Based on prior studies of child behavior problems, we anticipated three to five profiles would be identified that included stable (low stable, medium stable, high stable), increasing (low increasing), and/or decreasing (high decreasing) groups. Using these profiles, we then sought to test two specific hypotheses related to the prediction of risk profile membership. Specifically, we hypothesized that: (1) structured parenting would have a main effect on profile membership (increase the likelihood of membership in the low and the decreasing profiles), and (2) the genetic moderation of the effects of structured parenting previously identified cross-sectionally at 18 months in Leve et al. (2009) would extend to developmental profile membership across the following six and a half years. Structured parenting in toddlerhood, we predicted, would *reduce the likelihood* of a child’s membership in the high and increasing behavior problem groups for children with *higher levels of biological parent psychopathology*. Conversely, this same style of parenting *would increase the likelihood* of membership in the high and increasing behavior problem groups for children with *lower levels of biological parent psychopathology*.

## Method

### Participants

Participants included 361 adoptive parent, adopted child, and biological parent triads. Participants were recruited through adoption agencies in the Pacific Northwest, Mid-Atlantic, and Southwestern regions of the United States as part of a domestic adoption study. The sample included children with birthdates between January 2003 and June 2006. All children were placed for adoption with a nonrelative within 3 months of birth (*M* = 6.35 days, *SD* = 12.02 days; median = 2 days). Most families included male-female pairs of adoptive parents (*n* = 333), while a small number of families had same-sex adoptive parents (*n* = 20), were single mother families (*n* = 5), or had an unknown family constellation (*n* = 3). For parsimony, we refer to the primary adoptive caregiver as adoptive mother (97 % female), and the second adoptive caregiver as adoptive father (97% male). Forty-three percent of the children were females. Fifty-seven percent of the children were Non-Hispanic White, 11% were Black, 12% were Hispanic/Latino, 19% were more than one race, less than 1% were American Indian or Alaskan Native, less than 1% were Asian, less than 1% were Native Hawaiian or Pacific Islander, and less than 1% were unknown or not reported. Adoptive parent race and ethnicity for mothers and fathers, respectively, was 91%/90% non-Hispanic White, 4%/5% Black, 3%/2% Hispanic/Latino, 1%/1% American Indian, Native Hawaiian, Pacific Islander, or Asian, 1%/1% more than one race, and 1/%/1% were unknown or unreported. The mean age of the adoptive mothers and fathers at childbirth was 37.78 (*SD* = 5.5) and 38.39 (*SD* = 5.8), respectively. The mean age of the biological mother and biological father at childbirth was 24.12 (*SD* = 5.9) and 25.43 (*SD* = 7.18), respectively. At childbirth, nearly half of the adoptive parents were characterized as affluent and had annual gross household incomes that exceeded $100,000, and more than 70% of adoptive parents had completed a college education or higher. At childbirth, 62% of biological mothers and 46% of biological fathers had a household income of less than $20,000, and the majority did not have a college degree. For a more detailed description of sampling methods and participant characteristics, please refer to Leve et al. (2019).

All participants were assessed longitudinally through in-person assessments, online questionnaires, and telephone interviews. A range of measures was administered, as described below, across seven time points spanning from when the child was 9 months to 96 months (8 years old). All procedures were approved by the University of Oregon Institutional Review Board (Project No. 08082016.007; Title: The Early Growth and Development Study Pediatric Cohort) and informed consent was obtained from all adult participants; assent was obtained from child participants age 7 and older.

Participants were included regardless of missing data patterns, with latent profiles estimated using full information maximum likelihood, which can produce unbiased estimates as long as the data are missing at random conditional on the observed covariates (Enders & Bandalos, 2001; Enders, 2001). We assumed the data were missing at random conditional on the observed covariates. We tested this assumption with a series of t-tests that examined whether children who had missing data on each of the follow-up CBCL assessments differed on any model predictors, covariates, or child race from children who had complete CBCL data in the waves being compared. Of all the comparisons, only one significant difference was identified (openness, for children missing CBCL data at 27 months, *p* = .014); however, this comparison became nonsignificant when a Bonferroni correction for multiple comparisons was applied. Due to high levels of missingness for biological fathers, the primary models use biological mother data only, as is discussed in the data analytic approach. The percent of missing data for each variable is presented in Table 1.

### Measures

As the current report is an extension of Leve et al. (2009), we selected the same familial transmission measures as the prior report (e.g., the same measures of structured parenting and biological parent psychopathology), the same covariates, and the same measure of child behavior problems.

#### Child behavior problems

The total behavior problem T-Score from the CBCL was used at six assessment waves as an indicator of child behavior problems, which represents an age-based standardized score normed with a national sample with a mean of 50 and a standard deviation of 10 (Achenbach & Rescorla, 2001). The use of T-scores also helped accommodate the two versions of the CBCL that were used in this study (the age 1½–5 version and age 6–18 version, depending on the child age at the time of assessment). The CBCL is a widely used and well-validated questionnaire measure in assessing child social and emotional problem behaviors. Items are rated on a 3-point Likert scale, ranging from 0 (Not True) to 2 (Very True) based on the child’s behaviors. Cronbach’s alpha ranged from 0.94 to 0.96 across time points in the current study. The distributions at each time point were roughly normal. CBCL data were obtained from adoptive mothers and fathers at child ages 18, 27, 54, 72, 84, and 96 months. Replicating the approach taken by Leve et al. (2009), parent scores were averaged within each time point to address the primary research questions. Inter-parent correlations ranged from .35 to .52. When data were available for only one respondent within a time (i.e., 32% of the time and mostly missing for father reports), the corresponding score was used. The CBCL data served as the dependent variable for the latent profile analysis.

#### Biological parent psychopathology

Biological parent psychopathology was used as an index for the adoptees’ genetic risk for psychopathology. It was estimated by combining four measures for biological mothers (BM) and for biological fathers (BF): (a) alcohol, tobacco, and other drug use; (b) antisocial behavior; (c) depression; and (d) anxiety. Alcohol, tobacco, and other drug (ATOD) use was measured using a modified version of the Composite International Diagnostic Interview Short-Form Alcohol and Drug Dependence scales (Kessler et al., 1998). The modifications included a set of tobacco dependence questions and a lifetime use response frame. The indicators of lifetime problem use of alcohol, tobacco, marijuana, and other drugs were created, standardized, and combined to form a composite (BM α= .72, BF α= .71). The Elliott Social Behavior questionnaire evaluated antisocial behavior (Elliott et al., 1985). It is a self-report questionnaire containing 38 items (BM α= .88, BF α= .91). Depression was measured using 20 of the 21-item Beck Depression Inventory (Beck & Steer, 1987), with the suicidal ideation item not administered (BM α= .92, BF α= .89). Finally, anxiety was measured using the 21-item Beck anxiety scale (Beck & Steer, 1993; BM α= .90, BF α= .88). Each genetic risk variable was standardized separately for biological mothers and biological fathers.

#### Structured parenting

When children were 18 months, adoptive mother–child dyads participated in a structured 3-min clean-up task in their home in which the interviewer asked the mother to have her child clean up multiple toys and place each in its corresponding container. The parent was instructed not to assist in the cleaning, but only to instruct their child on the clean-up task. The task was later coded from digital recordings using the Parent-Child Free Play and Compliance Task Coding Manual (K. Pears and M. Ayers, unpublished coding manual, 2005), a real-time microsocial system that indicates the initiator, the initiator’s behavior (twodigit), and the recipient. When an observation code changes, a new code is entered. As in Leve et al. (2009), we used the “parental request” code, which measures the duration of time the parent spent making requests and commands of the child, with an explicit or implicit behavior change or a specific action desired of the child. We refer to this as structured parenting. Coded examples of structured parenting included, “Put the star in this hole,” and “Look at the shape of the piece, where does it go?” Fifteen percent of the tapes were coded by two independent coders; the average intercoder agreement on the behavior content code was 88% (overall κ= .71).

#### Covariates

Control variables included measures of openness in the adoption, biological mother prenatal ATOD use, and infant temperamental distress to limitations and fear. Biological mothers, biological fathers, adoptive mothers, and adoptive fathers individually rated the level of adoption openness (e.g., information about and contact with their counterpart) on a seven-point scale during infancy. Scores were standardized and a composite index was created; the interrater agreement was high (*r* range .66–.81; Ge et al., 2008). Biological mother prenatal ATOD use could confound estimates related to genetic risk, and thus was included as a control variable. Biological mothers self-reported their use of 10 substance classes (tobacco, alcohol, sedatives, tranquilizers, amphetamines, painkillers, inhalants, cocaine, heroin, and hallucinogens) using a pregnancy history calendar (Caspi, Moffitt, Thornton, et al. 1996). Data from this variable were collapsed to a dichotomous scale indicating “did (= 1) /did not (= 0) engage in prenatal drug use” (Cronbach α= .67). Finally, infant temperament was measured at child age 9 months using two subscales from the Infant Behavior Questionnaire (Rothbart, 1981). Specifically, adoptive mother and father report on the infant distress to limitations (20 items) and fear (16 items) subscales were used (α ranged from .71–.73), with each being composited between parent raters and converted to *z*-scores prior to analysis. Inclusion of the child temperament variables earlier in development (9 months) allowed us to consider the potential role that child behaviors may have had on our measure of maternal structured parenting at 18 months.

### Data analytic approach

We considered multiple analytic approaches, including developmental trajectory modeling and profile analysis. Inspection of the data indicated very low variance in the linear slope, and we received a model convergence warning when growth modeling approaches were employed. We therefore proceeded with a latent profile analysis approach.

We used a two-stage approach to analyses. In Stage 1, we estimated profiles of behavior problems using latent profile analysis (latent class analysis with continuous indicators), with each of the six CBCL time-points used as profile indicators. In Stage 2, following estimation of the latent profiles, multinomial logistic regression was used to examine if structured parenting and biological parent psychopathology, and the interaction between the two, predicted profile membership.

Across all our models, we used a variety of criteria to compare the model fit. For the profile analysis, we primarily relied on the BIC for statistical evidence of fit to the data, which has been shown to function well in simulation studies and should be preferred over AIC (Nylund et al., 2007). For the multinomial logistic regression analyses, we evaluated both the change in the model deviance, as indicated by a chi-square test, as well as changes in AIC. When interpreting information criteria (AIC or BIC), we used rules of thumb suggested by Burnham and Anderson (2004). Specifically, when the difference between the competing models was less than two, little evidence would support one model over the other. Differences between four and seven indicate “considerably less support” (p. 271) for the model with the higher value, while differences greater than ten provide “essentially no support” (p. 271) for the model with the higher value.

#### Stage 1: Profile analysis

Profile analysis estimates the probability of membership in each of *k* groups based on patterns of responses across the profile indicators (CBCL timepoints). The number of profiles to extract (*k*) is determined by theory and fit to the data. Conceptually, profile analysis (and all latent class approaches) estimates homogenous subpopulations within heterogeneous samples (Berlin et al., 2014). These approaches are therefore often referred to as “person-centered” analyses (e.g., Laursen & Hoff, 2006; Marsh et al., 2009), in contrast to the more typical “variable-centered” approaches (e.g., factor analysis). Generally, cases are assigned a profile based on the highest estimated probability of membership within that profile. For example, it is common for a two-profile solution to have “low” and “high” profiles. A child with universally high scores across the profile indicators would, in this example, have a high probability of being in the “high” group and be assigned as such, while having a corresponding low probability of being represented in the “low” group. Our analyses used the age-normed T-score to account for differences in the measures used across ages. Interpreting change over time in a T-score is difficult because of the age-based norming. However, in the current study we were interested in the patterns of scores over time, rather than the magnitude of change within individuals over time.

When determining the number of profiles to extract, we first estimated a set of candidate models ranging from 2 to 8 profiles. We then evaluated the extent to which models with the lowest BIC values aligned with theory. Balancing both the statistical and theoretical evidence, we arrived upon our final model, as described in the results section. Across all model fits, we estimated separate means and variances for each time point and allowed these means and variances to be different across profiles. However, to reduce the overall model complexity, covariances among the profile indicators (time points) were not estimated.

#### Stage 2: Multinomial logistic regression

After estimating the most likely profiles for each child, we estimated multinomial logistic regression models to evaluate how the log odds of membership in each of the estimated profiles depended upon structured parenting scores and biological parent psychopathology scores. Following the approach used by Leve et al. (2009), we entered predictor variables in blocks. In the first block, we included measures of openness in adoption, biological parent prenatal drug use, infant distress to limitations, and infant fear. This block of variables was entered primarily as control variables. In the second block, we included the main effects of structured parenting and biological parent psychopathology, which represented our environmental and genetic predictors, respectively. Finally, in the third block, we included the interaction between structured parenting and biological parent psychopathology.

Our primary model included psychopathology ratings for biological mothers only serving as the indicator of genetic risk, based on the higher rates of missing data for biological father psychopathology ratings. Following the fitting of the biological mother model, we fit a combined model that averaged the psychopathology ratings of the biological mother and biological father when data from both respondents were available and used the biological mother rating otherwise. The resulting variable was therefore a weighted composite, with more cases that had biological mother only ratings than cases that included both biological parents’ data. Roughly one-third of the cases represented an average of both biological parents (*n* = 100) while the remaining two-thirds (*n* = 213) of cases were represented by biological mother psychopathology ratings only. This exploratory analysis was conducted to examine if the risk profiles remained stable with the inclusion of both biological parents in the psychopathology ratings.

Latent profile analyses were estimated with *Mplus* (Muthén & Muthén, 1998–2017), via the *tidyLPA* package (Rosenberg, 2018) within the R statistical computing environment (R Core Team, 2018). Multinomial logistic regression analyses were conducted using the *nnet* package (Venables & Ripley, 2002). Data preparation was conducted using the *tidyverse* suite of packages (Wickham, 2017), which includes the *ggplot2* package (Wickham, 2009) that was used for all data displays.

## Results

Means, standard deviations, percent of missing cases, and the correlation matrix for all variables used in this study are presented in Table 1. Approximately 8–32% of cases on the CBCL were missing, which was handled with full-information maximum likelihood estimation. Among the CBCL time points, the correlation among the CBCL items generally followed a first-order autoregressive structure, where time points more closely spaced correlated higher than those spaced further apart.

### Stage 1: Profile analysis

**Figure 1.**
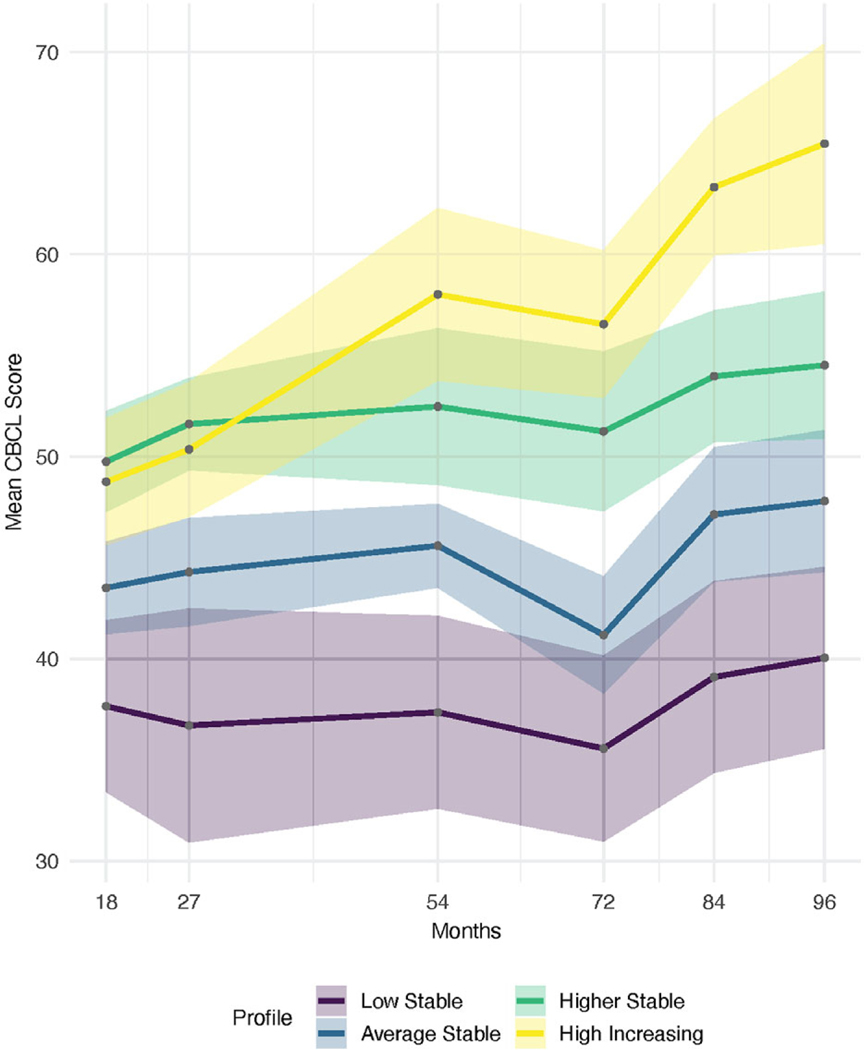
Mean scores over time across CBCL profiles. *Note*. CBCL = Child Behavior Checklist.

Models that included 5–8 estimated profiles did not converge to a proper solution and could not be estimated. Of the two-, three-, and four-profile solutions, the four-profile model displayed the best fit to the data, as indicated by BIC (6504.97, 6428.613, and 6426.794, respectively), although the BIC values were quite similar for the three- and four-profile solutions. From a theoretical perspective, the four-profile solution was also more consistent with that of other samples (Hill et al., 2006) and thus for both statistical and theoretical reasons, was retained as the final solution. Figure 1 displays the estimated means at each time point, along with 95% CIs for the four-profile solutions.

For the final four-profile model, the lowest (purple line in Figure 1; labeled *Low Stable*) and highest (yellow line, labeled *High Increasing*) profiles had the fewest number of children, with 39 and 35 children, or roughly 11% and 10% of the total sample, respectively. The lower group of the two middle profiles (blue line, labeled *Average Stable*) included the most children, with 177 children, or roughly 50% of the sample. Finally, the higher group of the two middle profiles included the remaining 102 children, or roughly 29% of the total sample (green line, labeled *Higher Stable*). Note that Figure 1 also implicitly displays the sample size through the CIs (shading around the line), as the size of the intervals depends primarily on sample size, with larger samples resulting in tighter intervals. Entropy for the model was 0.79, indicating adequate separation among groups. Table 2 displays the means and standard deviations for each profile.

### Stage 2: Multinomial logistic regression

Following the latent profile analyses, we fit multinomial logistic regression models to determine if measures of structured parenting or biological parent psychopathology would predict the most probable behavioral profile. We entered predictor variables block-wise, as described in the methods section. For all analyses, we used the group displaying the lowest behavioral problems over time as the reference group (the low stable group).

#### Biological mother model

The first model fit was fully unconditional, or an intercept-only model, representing the baseline probability of a child being classified in each profile. Following the unconditional model, we added our first block of predictor variables, which included openness in adoption, the dichotomous indicator of prenatal ATOD, and the standardized variables relating to infant temperamental distress to limitations and fear. Inclusion of these variables resulted in a significant reduction in the model deviance (*χ*^2^(12) = 35.55, *p* < .001). The AIC was also reduced substantially (AIC = −11.55), indicating support for the conditional model over the unconditional model. The conditional odds ratios of classification in each profile were 4.46, 2.32, and 1.22 for the *Average Stable, Higher Stable*, and *High Increasing*, as opposed to the *Low Stable* profile, respectively. These represented an overall reduced likelihood of being in either *Average Stable* or *Higher Stable*, and a slightly higher likelihood of being in the *High Increasing* profile, after controlling for the aforementioned variables. Note, however, that the significance did not change (probability of classification in the *High Increasing* profile was not significantly different from the *Low Stable* profile, *p* = .55). None of the variables in the first block individually significantly changed the odds of being in the *Average Stable* profile as opposed to the *Low Stable* profile. Children with higher scores on the distress to limitations variable were significantly more likely to be in the *Higher Stable* or *High Increasing* profiles, as opposed to the *Low Stable* profile. Specifically, a one-standard deviation increase in distress to limitations corresponded to children being 2.97 times more likely to be in the *Higher Stable* profile, and 2.52 times more likely to be in the *High Increasing* profile, as compared to the low profile.

The second block of variables included the addition of structured parenting and biological mother psychopathology, which did not result in a significant reduction in the model deviance (*χ*^2^(6) = 7.51, *p* = .28), while increasing AIC (AIC = 4.49). Neither of the variables significantly related to any behavioral profile. Finally, the third block included the addition of the interaction between structured parenting and biological mother psychopathology. The addition of the interaction did significantly reduce the overall model deviance, related to the model with the variables entered as main effects only (*χ*^2^= 9.78, *p* = .02). The AIC was marginally reduced (AIC = −3.78). Table 3 reports the coefficients, SEs, ORs, and 95% CIs for the final model; Supplementary Tables S1–S3 present this information for the earlier blocks in the model.

**Figure 2.**
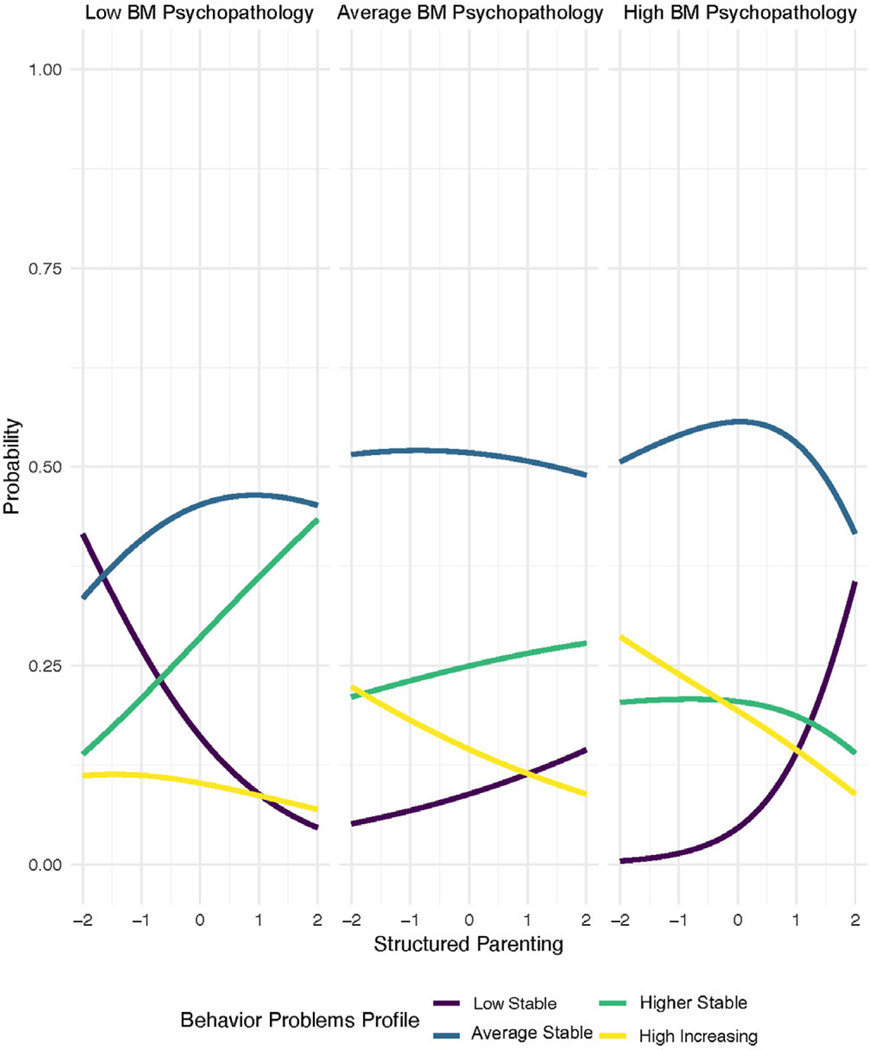
Predicted probability of CBCL profile membership by structured parenting and biological mother psychopathology.

As can be seen in Table 3, the interaction was significant in all models. Figure 2 displays these interactions visually. The figure is paneled by biological mother psychopathology scores, with those on the left being one or more standard deviations below the sample mean, those in the middle being within one standard deviation of the sample mean, and those on the right being one or more standard deviations higher than the sample mean (representing low, average, and high genetic risk). Each line in the plot represents a CBCL behavior problem profile, while the *y*-axis represents the probability that children will be classified in the corresponding profile. Structured parenting scores are represented by the *x*-axis. Thus, the plot displays how the probability of being classified in each of the CBCL behavioral profiles changes by structured parenting scores within a group of children with similar biological mother psychology scores (e.g., genetic risk indicators). For children with low biological mother psychopathology (the first panel in Figure 2), their likelihood of being in the *Low Stable* profile decreased as structured parenting increased, while their likelihood of being in the *Higher Stable* profile increased. For children with high biological mother psychopathology (the third panel in Figure 2), their likelihood of being in the *Low Stable* profile increased (rather than decreased) as structured parenting scores increased, while their likelihood of being in either the *Higher Stable* or *High Increasing* profiles decreased (particularly *High Increasing*).

#### Combined biological parent model

Overall, the results of the combined model (biological mother/biological father combined scores) were largely similar to the results of the biological mother only model. The second block of variables, which included the same structured parenting variable and the biological parent psychopathology composite, again did not result in a significant reduction in the model deviance (*χ*^2^(6) = 5.79, *p* = .45) and the model AIC again increased (AIC = 6.21). Unlike the biological-mother only model, however, the addition of the interaction did not result in significant reduction in the model deviance (*χ*^2^(3) = 7.80, *p* = .05). The model AIC was very marginally reduced (AIC = −1.80), suggesting the models were roughly equivalent. Despite the model not fitting significantly better overall, the individual interaction coefficients were again uniformly significant. The estimates themselves were also all very similar, generally within 0.03 points. Although not displayed, Figure 2 was replicated for this analysis and was nearly indistinguishable visually from the model fit with biological-mother only psychopathology ratings. See Supplementary Tables S4–S6 for the multinomial logistic regression models for the combined biological parent models.

## Discussion

To our knowledge, this is the first study to examine familial transmission by investigating whether a genetically moderated parenting effect identified in toddlerhood extends longitudinally to developmental profiles of child behavior problems. Unlike studies of genetically related parents and children where genetic and environmental influences are fully confounded, the adoption study allowed us to test whether parenting effects on child behavior problem profiles were moderated by genetic influences passed to the adoptee from their biological parent. Using data from a longitudinal adoption study, we first conducted a profile analysis with six waves of CBCL data that spanned from ages 18 months to 8 years. Results suggested a four-profile model that included a low stable group, an average stable group, a higher stable group, and a high increasing group, with children in the latter two groups showing clinically meaningful problems at some time points. Multinomial logistic regression models tested whether structured parenting, biological parent psychopathology, and their interaction were predictive of behavior problem profiles, and suggested a genetically moderated parenting effect, described in more detail below. We first discuss the behavior problem profiles that were identified.

### Behavior problem profiles from 18 months to age 8

The current study identified four distinct patterns of behavior problems from age 18 months to age 8. The majority of children were in a group that showed average levels of behavior problems (relative to population norms) that were stable over time. In addition, some children were in a profile that showed higher yet stable behavior problems over time, and a smaller number of children were in a group that showed stable and low behavior problems over time. Perhaps most interesting from a prevention standpoint is the small group of children in this study (*n* = 35) who showed high increasing behavior problems over time, and in some cases at mean levels that fell in a borderline clinical range. As discussed later, the results of the logistic regression suggest that coaching parents to use more structured parenting techniques during toddlerhood may be a window of opportunity to prevent the escalation of behavior problems for this group, when children are at higher genetic susceptibility for problems. In addition, our results indicated that children rated higher on distress to limitations in infancy were more likely to be in the higher stable or the high increasing groups, as compared to the low stable group, suggesting consideration of child temperament as an early screener for participation in prevention efforts.

Although the identification of four groups is not surprising and generally replicates others’ work examining developmental trajectories of behavior problems (e.g., Broidy et al., 2003; Shaw et al., 2003), we did not anticipate that overall, most children would show stable or increasing levels of behavior problems over time and no group of children would be identified who showed a decreasing pattern of behavior problems. As discussed in the introduction, many developmental studies of behavior problem trajectories from age 18 months to age 8 years show a decreasing level of behavior problems over time, particularly for overt externalizing problems (e.g., Williams et al., 2009). Some researchers have found that internalizing behavior problems begin to increase by age 5 or 6 (e.g., Gilliom & Shaw, 2004; Liu et al., 2020), and because we used the CBCL’s total behavior problem score, the use of both externalizing and internalizing items may partially explain our results, particularly given the final age of assessment in the current study (age 8). Additionally, it is important to note that our analysis was group profile membership based, not latent growth modeling based. Preliminary models were also estimated using growth mixture modeling (see Jung & Wickrama, 2008). However, CBCL T-Scores are not designed to measure “growth” in the sense of expected linear gains (or losses) over time. Indeed, the T-Score, used in this study, was designed to have the same mean at each time point, following typical normative trajectories. The results of the growth mixture models made little theoretical sense, with all but a few participants (~5) classified in a single class. We therefore conducted latent profile analysis in the current study to allow us to inspect patterns of behaviors across the time points without imposing any constraints about the nature of change over time (i.e., we evaluated patterns across time, rather than change over time). As such, it is important not to over-interpret the visual appearance of increasing behavior problems scores in Figure 1 based on the analytic approach used.

### Genetic moderation of parenting effects

A primary purpose of the current study was to test whether structured parenting at 18 months had a long-term predictive association with children’s behavior problem profiles that was conditioned by the child’s genetic risk for psychopathology, as measured by their biological parents’ psychopathology. The results extend the cross-over interaction pattern that was identified cross-sectionally at 18 months of age, whereby structured parenting was associated with fewer behavior problems for children with higher genetic risk, and with more behavior problems for children with lower genetic risk (Leve et al., 2009). Specifically, in the current study, for children whose biological parents had higher psychopathology, the odds of the child being in the low stable group increased when structured parenting was higher. Conversely, the child’s odds of being in the higher stable or the high increasing group decreased when maternal structured parenting was higher. This promising news has relevance for the design of prevention programs, as is discussed in the next section. Similar to the Leve et al. (2009) cross-sectional study, the current study also found that when a child’s biological parents had lower psychopathology, higher levels of structured parenting by the adoptive parent had the opposite effect as it did for a child whose biological parents had higher psychopathology. Specifically, when the biological parents had lower psychopathology, the odds of the child being in the low stable CBCL group went down when levels of structured parenting were higher. In fact, these children were more likely to be in the higher stable group. This finding suggested the powerful role that structured parenting during toddlerhood may play in preventing or promoting a higher or an increasing profile of behavior problems to age 8, as a function of the child’s genetic risk for psychopathology. In other words, what is risk reducing for one child can be risk promotive for another child, based on the child’s genetic make-up.

### Prevention implications

A core element of translating basic science research into prevention practice is to examine whether effects are persistent or durable prior to translation. If, for example, the genetically moderated parenting effects evidenced in toddlerhood (Leve et al., 2009) did not sustain and only predicted children’s behavior problems at a single and a rather early time point (toddlerhood), it would not be warranted to use knowledge of genetic background to guide preventive intervention; there would be limited developmental relevance of structured parenting to the prevention of behavior problems for a selected group of children as a function of their genetic background. On the other hand, if genetically moderated effects are sustained later in development (as they were in the current study) and we learn that structured parenting in toddlerhood leads to clinically-meaningful patterns of behavior problems from toddlerhood to age 8 that are conditioned by genetic factors, such findings can provide novel information to consider in studies designed to leverage genetic information to guide the identification of children into prevention studies and optimally, tailor specific parenting interventions to specific contextual and genetic backgrounds. In particular, when children are at elevated genetic risk for behavior problems, the ability to leverage interventions that coach parents to increase their use of structured parenting in toddlerhood could ultimately reduce or eliminate the group of children who have higher or high increasing profile of behavior problems, as was seen in the current study. Although replication efforts are needed, the current study adds to the body of literature showing the long-term beneficial effects of structured parenting in toddlerhood, but with the caveat that these benefits may only be present for children with higher genetic risk and may in fact be detrimental when a child has lower genetic risk. Although most psychosocial prevention and intervention studies that enroll children into parenting support programs select families whose children are at elevated risk for behavior problems based on multiple familial indicators, the current results suggest that “one size may not fit all” in prescribing effecting parenting strategies depending on variation in genetic risk. As measurement of parent genetic risk is often not feasible in prevention studies or clinical practice, the findings also suggest that as part of the screening process for treating early child problem behavior, parents are screened for markers of psychopathology that demonstrate heritability (e.g., depression, anxiety, personality disorders).

The current study also highlights the role of the child’s temperamental distress to limitations during infancy in predicting behavior problem profiles. Specifically, children rated as higher on distress to limitation in infancy were more likely to be in the high stable or the high increasing group on the CBCL (relative to the low stable group), although caution is warranted in interpreting this association given that it could have arisen from shared method variance (both measures were adoptive parent report). Nonetheless, this finding may suggest that temperamental distress to limitations could be used as a screening mechanism in infancy to select families where additional parenting and family support programs during toddlerhood could coach parents on effective parenting strategies for their child that could prevent a high and increasing pattern of behavior problems from evolving. Guided by the work of McClowry (McClowry & Collins, 2012; McClowry et al., 2008), these findings suggest that additional efforts to develop and test temperament-based interventions are needed. They also suggest the need for additional investigation into reciprocal associations between temperament, parenting, and behavior problems to help identify developmental periods when interventions that modify one of these behaviors could have downstream effects on other behaviors (e.g., when changes in temperament may lead to changes in parenting and when changes in parenting may lead to changes in behavior problems).

### Limitations

Several limitations of the current study should be noted when interpreting and applying the current findings. First, our study only included families involved in infant domestic adoptions in the United States. This design attribute was essential for testing the genetic moderation of parenting in the manner we approached our research questions, but it comes with some limitations. Specifically, as detailed in the methods section, most adoptive parents in this study had high incomes, advanced educational degrees, and self-identified as non-Hispanic White. It is unknown whether structured parenting would have the same effects on children’s behavior problem profiles in families with more socio-economic and racial diversity. In addition, we used adoptive parent report on CBCL as our sole outcome measure. Although we aggregated scores across both adoptive parent reporters (where two reports were available) and we used a multimethod approach such that the parenting variable was coded from an observational task and the biological parent psychopathology variable was generated from biological parents, the reliance on adoptive parent report of child behavior is a limitation. Third, because our focus was on the sustained impact of structured parenting during toddlerhood, this report does not include measures of parenting or contextual influences on child behavior problems (e.g., peer influences) later in childhood that are known to predict behavior problem profiles into middle childhood and may mediate or moderate the current pattern of findings. Fourth, although we noted earlier the potential prevention implications based on the genetically moderated effects of structured parenting on child behavior problems, we have not provided a roadmap for how one would conduct such prevention services in the field. For example, community-based practitioners may not have access to the genetic background of the children they are serving. Nonetheless, clinicians and practitioners sometimes do have access to family history of psychopathology, which can be used as a proxy for genetic risk in biological families, just as it was in the current adoption study, and many prevention studies routinely screen for parental depression, anxiety, substance use, and/or history of antisociality. Although not a pure measure of genetic influences, in the absence of genetic testing and genetic history, assessing family history of psychopathology is one method that practitioners could use to better understand the risk and protective tendencies that children may have inherited, and take us one step closer to designing and implementing genetically informed prevention services that are better tailored to an individual child’s needs and growth areas.

Overall, the current study is the first to demonstrate how the child’s genetic make-up can alter whether a specific type of parenting in toddlerhood has a promotive or a suppressive effect on children’s behavior problems throughout childhood, across multiple stages of developmental (spanning 6.5 years). The same type of parenting that is beneficial for some children can be risk-enhancing to other children. This new insight, made possible through the use of a genetically sensitive adoption design, adds to our understanding of familial transmission of psychopathology and suggests one avenue whereby preventive interventions might collectively leverage information about the child’s genetics and parenting to better tailor parenting support programs.

## Supplementary Material

1

## Figures and Tables

**Table 1.** Correlations and descriptive statistics for all variables

Variable	1	2	3	4	5	6	7	8	9	10	11	12	13
1. Openness													
2. Pren ATOD	0.08												
3. DTL	0.01	0.03											
4. Fear	−0.14*	−0.05	0.39***										
5. Str Parenting	0.04	0.00	−0.03	−0.02									
6. CBCL 18	0.06	0.03	0.39***	0.17**	−0.12*								
7. CBCL 27	−0.02	0.03	0.31***	0.07	−0.10	0.72***							
8. CBCL 54	−0.04	−0.05	0.16**	0.12*	−0.10	0.53***	0.56***						
9. CBCL 72	−0.08	0.07	0.17**	0.06	−0.01	0.50***	0.59***	0.74***					
10. CBCL 84	0.04	0.00	0.17**	0.10	−0.03	0.42***	0.47***	0.64***	0.67***				
11. CBCL 96	−0.01	−0.01	0.03	−0.02	−0.07	0.40***	0.42***	0.63***	0.63***	0.77***			
12. BF Psychop	0.07	0.15	−0.03	−0.14	−0.08	−0.08	−0.08	0.01	0.01	−0.01	0.02		
13. BM Psychop	0.00	0.34***	−0.02	−0.06	−0.01	0.01	0.02	−0.01	0.00	0.07	0.04	0.23*	
Mean	−0.02	0.56	0.00	−0.01	0.00	45.07	46.12	48.17	45.16	49.70	50.83	−0.02	0.00
SD	0.90	0.50	0.90	0.91	1.00	6.48	7.09	7.83	8.34	8.08	9.18	0.73	0.76
Percent missing	0.00	0.01	0.03	0.06	0.07	0.08	0.12	0.24	0.24	0.29	0.32	0.69	0.00

*Note.* Pren ATOD = prenatal alcohol, tobacco, and other drug use; DTL = infant distress to limitations; Str Parenting = structured parenting; CBCL, with the corresponding child age in months; BF Psychop = biological father psychopathology; BM psychop = biological mother psychopathology.

* *p* < .05,

** *p* < .01.

*** *p* < .001.

**Table 2.** Means and standard deviations for CBCL behavior problem T-scores at each time point and profile

Profile	Months	Mean	95% CI		*SD*	95% CI	
			Lower bound	Upper bound		Lower bound	Upper bound
1	18	37.65	33.41	41.89	2.17	1.46	2.52
1	27	36.70	30.91	42.48	2.25	0.00	2.70
1	54	37.35	32.59	42.11	2.18	1.86	2.40
1	72	35.56	30.96	40.15	2.13	0.00	2.58
1	84	39.09	34.36	43.83	2.35	2.00	2.59
1	96	40.04	35.55	44.53	2.57	0.00	3.09
2	18	48.75	45.61	51.89	2.70	2.29	2.98
2	27	50.35	47.02	53.68	2.81	2.30	3.14
2	54	58.01	53.75	62.28	2.91	1.77	3.40
2	72	56.54	52.89	60.19	2.84	0.00	3.39
2	84	63.32	59.93	66.71	1.97	1.57	2.22
2	96	65.47	60.51	70.43	2.33	1.51	2.71
3	18	49.75	47.26	52.24	2.15	1.89	2.34
3	27	51.60	49.32	53.88	2.31	2.09	2.48
3	54	52.47	48.60	56.34	2.10	1.68	2.35
3	72	51.24	47.29	55.18	1.97	1.37	2.27
3	84	53.96	50.71	57.22	1.95	1.73	2.11
3	96	54.51	50.87	58.15	2.34	1.91	2.61
4	18	43.50	41.22	45.78	2.18	2.00	2.33
4	27	44.28	41.61	46.95	2.06	1.84	2.22
4	54	45.58	43.51	47.65	2.17	1.94	2.35
4	72	41.17	38.29	44.06	2.18	1.99	2.34
4	84	47.13	43.81	50.44	2.26	1.94	2.48
4	96	47.79	44.28	51.30	2.46	2.15	2.69

*Note*. 1 = Low stable group (*n* = 39); 2 = high increasing group (*n* = 35); 3 = higher stable group (*n* = 102); 4 = average stable group (*n* = 177).

**Table 3.** Final multinomial logistic regression model coefficients: biological mothers

Level	Parameter	OR	*SE*	CI		*p*
				Lower bound	Upper bound	
Ave stable	Intercept	5.87	0.33	3.05	11.30	.00
Ave stable	Openness	0.87	0.23	0.56	1.36	.55
Ave stable	Pren ATOD	1.14	0.41	0.51	2.57	.75
Ave stable	DTL	1.30	0.26	0.78	2.18	.31
Ave stable	Fear	0.90	0.24	0.56	1.45	.67
Ave stable	Str Parenting	0.76	0.22	0.49	1.18	.22
Ave stable	BM Psy	2.08	0.40	0.95	4.58	.07
Ave stable	Str Par × BM Psy	0.41	0.32	0.22	0.76	.01
Higher stable	Intercept	2.83	0.36	1.39	5.75	.00
Higher stable	Openness	0.99	0.25	0.61	1.61	.98
Higher stable	Pren ATOD	1.27	0.45	0.52	3.07	.60
Higher stable	DTL	2.85	0.28	1.63	4.98	.00
Higher stable	Fear	0.84	0.26	0.50	1.41	.51
Higher stable	Str Parenting	0.83	0.24	0.52	1.32	.43
Higher stable	BM Psy	1.59	0.43	0.69	3.66	.28
Higher stable	Str Par × BM Psy	0.36	0.36	0.18	0.72	.00
High increase	Intercept	1.64	0.40	0.75	3.57	.21
High increase	Openness	0.92	0.29	0.52	1.64	.77
High increase	Pren ATOD	0.54	0.55	0.18	1.57	.26
High increase	DTL	2.51	0.34	1.30	4.87	.01
High increase	Fear	0.86	0.31	0.46	1.59	.63
High increase	Str Parenting	0.61	0.28	0.35	1.06	.08
High increase	BM Psy	2.58	0.45	1.06	6.29	.04
High increase	Str Par × BM Psy	0.40	0.40	0.18	0.86	.02

*Note*. All coefficients are relative to the *Low Stable* profile (the reference group). Ave = Average; Pren ATOD = prenatal alcohol, tobacco, and other drug use; DTL = infant distress to limitations; Str Parenting = structured parenting; BM Psy = biological mother psychopathology. Str Par = structured parenting.
