# Supplementary material for: Developmental profiles of child behavior problems from 18 months to 8 years: The protective effects of structured parenting vary by genetic risk
Source: Dev Psychopathol. Author manuscript; Available in PMC 2024 Feb 5. (PMC9899296; doi:10.1017/S0954579422000839)
Supplement: 1 [file NIHMS1823238-supplement-1.docx]

**Supplemental Materials**

Included in this online supplement are the results for each block in the multinomial logistic regression models for the biological mothers (the manuscript presents the final model only). In addition, we have included the results for each block in the multinomial logistic regression models for the combined biological mother/biological father models (not presented in the manuscript).

Table S1. *Multinomial Logistic Regression Model Coefficients: Biological Mothers -* *Block 1*

Table S2. *Multinomial Logistic Regression Model Coefficients: Biological Mothers -* *Block 2*

Table S3. *Multinomial Logistic Regression Model Coefficients: Biological Mothers -* *Block 3 (Final Model as Displayed in Manuscript Table 3).*

Table S4. *Multinomial Logistic Regression Model Coefficients: Biological Mother/Biological Father Combined -* *Block 1*

Table S5. *Multinomial Logistic Regression Model Coefficients: Biological Mother/Biological Father Combined -* *Block 2*

Table S6. *Multinomial Logistic Regression Model Coefficients: Biological Mother/Biological Father Combined -* *Block 3*

**Table S1**

*Multinomial Logistic Regression Model Coefficients: Biological Mothers -* *Block 1*

| y.level | term | estimate | std.error | statistic | p.value | conf.low | conf.high |
| --- | --- | --- | --- | --- | --- | --- | --- |
| Average Stable | (Intercept) | 1.494 | 0.294 | 5.082 | 0 | 0.918 | 2.07 |
| Average Stable | openness | -0.08 | 0.222 | -0.36 | 0.719 | -0.516 | 0.356 |
| Average Stable | prenatal_drug | 0.281 | 0.387 | 0.727 | 0.467 | -0.477 | 1.04 |
| Average Stable | distresslim | 0.287 | 0.259 | 1.108 | 0.268 | -0.22 | 0.794 |
| Average Stable | distressnov | -0.067 | 0.24 | -0.28 | 0.78 | -0.537 | 0.403 |
| Higher Stable | (Intercept) | 0.843 | 0.322 | 2.62 | 0.009 | 0.212 | 1.474 |
| Higher Stable | openness | 0.058 | 0.242 | 0.24 | 0.81 | -0.417 | 0.533 |
| Higher Stable | prenatal_drug | 0.262 | 0.425 | 0.617 | 0.537 | -0.571 | 1.096 |
| Higher Stable | distresslim | 1.089 | 0.282 | 3.856 | 0 | 0.536 | 1.643 |
| Higher Stable | distressnov | -0.131 | 0.26 | -0.502 | 0.616 | -0.641 | 0.38 |
| High Increasing | (Intercept) | 0.198 | 0.361 | 0.548 | 0.584 | -0.51 | 0.905 |
| High Increasing | openness | -0.026 | 0.288 | -0.09 | 0.928 | -0.59 | 0.538 |
| High Increasing | prenatal_drug | -0.332 | 0.508 | -0.654 | 0.513 | -1.327 | 0.663 |
| High Increasing | distresslim | 0.925 | 0.329 | 2.807 | 0.005 | 0.279 | 1.57 |
| High Increasing | distressnov | -0.131 | 0.311 | -0.422 | 0.673 | -0.74 | 0.478 |

Note. distresslim = Distress to Limitation. distressnov = Distress to Novelty.

**Table S2**

*Multinomial Logistic Regression Model Coefficients: Biological Mothers -* *Block 2*

| y.level | term | estimate | std.error | statistic | p.value | conf.low | conf.high |
| --- | --- | --- | --- | --- | --- | --- | --- |
| Average Stable | (Intercept) | 1.606 | 0.312 | 5.144 | 0 | 0.994 | 2.218 |
| Average Stable | openness | -0.059 | 0.222 | -0.265 | 0.791 | -0.495 | 0.377 |
| Average Stable | prenatal_drug | 0.125 | 0.41 | 0.305 | 0.761 | -0.678 | 0.928 |
| Average Stable | distresslim | 0.277 | 0.257 | 1.079 | 0.281 | -0.226 | 0.78 |
| Average Stable | distressnov | -0.051 | 0.241 | -0.213 | 0.831 | -0.524 | 0.421 |
| Average Stable | struc_parenting | -0.211 | 0.206 | -1.027 | 0.304 | -0.614 | 0.192 |
| Average Stable | bm_psychopathology | 0.351 | 0.321 | 1.093 | 0.274 | -0.278 | 0.98 |
| Higher Stable | (Intercept) | 0.883 | 0.341 | 2.587 | 0.01 | 0.214 | 1.552 |
| Higher Stable | openness | 0.073 | 0.241 | 0.301 | 0.764 | -0.4 | 0.545 |
| Higher Stable | prenatal_drug | 0.226 | 0.448 | 0.505 | 0.613 | -0.652 | 1.105 |
| Higher Stable | distresslim | 1.068 | 0.279 | 3.824 | 0 | 0.521 | 1.616 |
| Higher Stable | distressnov | -0.126 | 0.261 | -0.483 | 0.629 | -0.637 | 0.385 |
| Higher Stable | struc_parenting | -0.117 | 0.223 | -0.526 | 0.599 | -0.554 | 0.319 |
| Higher Stable | bm_psychopathology | 0.088 | 0.35 | 0.25 | 0.803 | -0.599 | 0.774 |
| High Increasing | (Intercept) | 0.33 | 0.379 | 0.871 | 0.384 | -0.413 | 1.073 |
| High Increasing | openness | -0.01 | 0.291 | -0.036 | 0.972 | -0.58 | 0.559 |
| High Increasing | prenatal_drug | -0.621 | 0.543 | -1.144 | 0.253 | -1.686 | 0.443 |
| High Increasing | distresslim | 0.934 | 0.333 | 2.803 | 0.005 | 0.281 | 1.587 |
| High Increasing | distressnov | -0.1 | 0.313 | -0.319 | 0.749 | -0.713 | 0.513 |
| High Increasing | struc_parenting | -0.425 | 0.263 | -1.617 | 0.106 | -0.94 | 0.09 |
| High Increasing | bm_psychopathology | 0.573 | 0.381 | 1.504 | 0.133 | -0.174 | 1.321 |

Note. distresslim = Distress to Limitation. distressnov = Distress to Novelty. struc_parenting = Structured Parenting. bm_psychopathology = Biological Mother Psychopathology.

**Table S3**

*Multinomial Logistic Regression Model Coefficients: Biological Mothers -* *Block 3 (Final Model as Displayed in Manuscript Table 3).*

| y.level | term | estimate | std.error | statistic | p.value | conf.low | conf.high |
| --- | --- | --- | --- | --- | --- | --- | --- |
| Average Stable | (Intercept) | 1.77 | 0.334 | 5.293 | 0 | 1.114 | 2.425 |
| Average Stable | openness | -0.135 | 0.228 | -0.593 | 0.553 | -0.581 | 0.311 |
| Average Stable | prenatal_drug | 0.131 | 0.414 | 0.315 | 0.753 | -0.681 | 0.943 |
| Average Stable | distresslim | 0.264 | 0.262 | 1.007 | 0.314 | -0.25 | 0.777 |
| Average Stable | distressnov | -0.103 | 0.242 | -0.425 | 0.671 | -0.577 | 0.371 |
| Average Stable | struc_parenting | -0.274 | 0.223 | -1.227 | 0.22 | -0.711 | 0.164 |
| Average Stable | bm_psychopathology | 0.734 | 0.402 | 1.825 | 0.068 | -0.054 | 1.522 |
| Average Stable | struc_parenting:bm_psychopathology | -0.899 | 0.32 | -2.807 | 0.005 | -1.528 | -0.271 |
| Higher Stable | (Intercept) | 1.039 | 0.362 | 2.87 | 0.004 | 0.33 | 1.749 |
| Higher Stable | openness | -0.005 | 0.247 | -0.021 | 0.983 | -0.489 | 0.478 |
| Higher Stable | prenatal_drug | 0.236 | 0.452 | 0.522 | 0.601 | -0.65 | 1.123 |
| Higher Stable | distresslim | 1.048 | 0.285 | 3.676 | 0 | 0.489 | 1.606 |
| Higher Stable | distressnov | -0.174 | 0.262 | -0.662 | 0.508 | -0.687 | 0.34 |
| Higher Stable | struc_parenting | -0.191 | 0.24 | -0.795 | 0.427 | -0.662 | 0.28 |
| Higher Stable | bm_psychopathology | 0.464 | 0.425 | 1.091 | 0.275 | -0.37 | 1.298 |
| Higher Stable | struc_parenting:bm_psychopathology | -1.027 | 0.358 | -2.872 | 0.004 | -1.728 | -0.326 |
| High Increasing | (Intercept) | 0.495 | 0.397 | 1.247 | 0.212 | -0.283 | 1.274 |
| High Increasing | openness | -0.086 | 0.295 | -0.29 | 0.772 | -0.663 | 0.492 |
| High Increasing | prenatal_drug | -0.62 | 0.546 | -1.136 | 0.256 | -1.691 | 0.45 |
| High Increasing | distresslim | 0.921 | 0.337 | 2.73 | 0.006 | 0.26 | 1.583 |
| High Increasing | distressnov | -0.151 | 0.314 | -0.481 | 0.63 | -0.767 | 0.465 |
| High Increasing | struc_parenting | -0.493 | 0.279 | -1.765 | 0.078 | -1.04 | 0.054 |
| High Increasing | bm_psychopathology | 0.949 | 0.454 | 2.09 | 0.037 | 0.059 | 1.839 |
| High Increasing | struc_parenting:bm_psychopathology | -0.925 | 0.396 | -2.335 | 0.02 | -1.701 | -0.149 |

Note. distresslim = Distress to Limitation. distressnov = Distress to Novelty. struc_parenting = Structured Parenting. bm_psychopathology = Biological Mother Psychopathology.

**Table S4**

*Multinomial Logistic Regression Model Coefficients: Biological Mother/Biological Father Combined -* *Block 1*

| y.level | term | estimate | std.error | statistic | p.value | conf.low | conf.high |
| --- | --- | --- | --- | --- | --- | --- | --- |
| Average Stable | (Intercept) | 1.494 | 0.294 | 5.082 | 0 | 0.918 | 2.07 |
| Average Stable | openness | -0.08 | 0.222 | -0.36 | 0.719 | -0.516 | 0.356 |
| Average Stable | prenatal_drug | 0.281 | 0.387 | 0.727 | 0.467 | -0.477 | 1.04 |
| Average Stable | distresslim | 0.287 | 0.259 | 1.108 | 0.268 | -0.22 | 0.794 |
| Average Stable | distressnov | -0.067 | 0.24 | -0.28 | 0.78 | -0.537 | 0.403 |
| Higher Stable | (Intercept) | 0.843 | 0.322 | 2.62 | 0.009 | 0.212 | 1.474 |
| Higher Stable | openness | 0.058 | 0.242 | 0.24 | 0.81 | -0.417 | 0.533 |
| Higher Stable | prenatal_drug | 0.262 | 0.425 | 0.617 | 0.537 | -0.571 | 1.096 |
| Higher Stable | distresslim | 1.089 | 0.282 | 3.856 | 0 | 0.536 | 1.643 |
| Higher Stable | distressnov | -0.131 | 0.26 | -0.502 | 0.616 | -0.641 | 0.38 |
| High Increasing | (Intercept) | 0.198 | 0.361 | 0.548 | 0.584 | -0.51 | 0.905 |
| High Increasing | openness | -0.026 | 0.288 | -0.09 | 0.928 | -0.59 | 0.538 |
| High Increasing | prenatal_drug | -0.332 | 0.508 | -0.654 | 0.513 | -1.327 | 0.663 |
| High Increasing | distresslim | 0.925 | 0.329 | 2.807 | 0.005 | 0.279 | 1.57 |
| High Increasing | distressnov | -0.131 | 0.311 | -0.422 | 0.673 | -0.74 | 0.478 |

Note. distresslim = Distress to Limitation. distressnov = Distress to Novelty.

**Table S5**

*Multinomial Logistic Regression Model Coefficients: Biological Mother/Biological Father Combined -* *Block 2*

| y.level | term | estimate | std.error | statistic | p.value | conf.low | conf.high |
| --- | --- | --- | --- | --- | --- | --- | --- |
| Average Stable | (Intercept) | 1.561 | 0.307 | 5.083 | 0 | 0.959 | 2.163 |
| Average Stable | openness | -0.065 | 0.223 | -0.293 | 0.769 | -0.502 | 0.371 |
| Average Stable | prenatal_drug | 0.187 | 0.409 | 0.458 | 0.647 | -0.615 | 0.989 |
| Average Stable | distresslim | 0.272 | 0.257 | 1.06 | 0.289 | -0.231 | 0.775 |
| Average Stable | distressnov | -0.055 | 0.241 | -0.229 | 0.819 | -0.528 | 0.417 |
| Average Stable | struc_parenting | -0.203 | 0.204 | -0.996 | 0.319 | -0.604 | 0.197 |
| Average Stable | psychopathology | 0.216 | 0.32 | 0.675 | 0.5 | -0.411 | 0.842 |
| Higher Stable | (Intercept) | 0.853 | 0.336 | 2.536 | 0.011 | 0.194 | 1.512 |
| Higher Stable | openness | 0.073 | 0.242 | 0.302 | 0.763 | -0.401 | 0.547 |
| Higher Stable | prenatal_drug | 0.267 | 0.447 | 0.596 | 0.551 | -0.61 | 1.143 |
| Higher Stable | distresslim | 1.066 | 0.28 | 3.814 | 0 | 0.518 | 1.614 |
| Higher Stable | distressnov | -0.13 | 0.261 | -0.499 | 0.618 | -0.643 | 0.382 |
| Higher Stable | struc_parenting | -0.115 | 0.222 | -0.518 | 0.604 | -0.55 | 0.32 |
| Higher Stable | psychopathology | -0.017 | 0.352 | -0.048 | 0.961 | -0.706 | 0.672 |
| High Increasing | (Intercept) | 0.276 | 0.375 | 0.736 | 0.462 | -0.459 | 1.011 |
| High Increasing | openness | -0.023 | 0.29 | -0.079 | 0.937 | -0.592 | 0.546 |
| High Increasing | prenatal_drug | -0.525 | 0.54 | -0.973 | 0.331 | -1.583 | 0.533 |
| High Increasing | distresslim | 0.928 | 0.333 | 2.785 | 0.005 | 0.275 | 1.581 |
| High Increasing | distressnov | -0.107 | 0.313 | -0.342 | 0.733 | -0.72 | 0.506 |
| High Increasing | struc_parenting | -0.412 | 0.262 | -1.573 | 0.116 | -0.925 | 0.101 |
| High Increasing | psychopathology | 0.398 | 0.388 | 1.026 | 0.305 | -0.363 | 1.159 |

Note. distresslim = Distress to Limitation. distressnov = Distress to Novelty. struc_parenting = Structured Parenting. psychopathology = Biological Father Psychopathology.

**Table S6**

*Multinomial Logistic Regression Model Coefficients: Biological Mother/Biological Father Combined -* *Block 3*

| y.level | term | estimate | std.error | statistic | p.value | conf.low | conf.high |
| --- | --- | --- | --- | --- | --- | --- | --- |
| Average Stable | (Intercept) | 1.651 | 0.32 | 5.164 | 0 | 1.025 | 2.278 |
| Average Stable | openness | -0.133 | 0.228 | -0.583 | 0.56 | -0.579 | 0.313 |
| Average Stable | prenatal_drug | 0.215 | 0.411 | 0.524 | 0.6 | -0.591 | 1.021 |
| Average Stable | distresslim | 0.254 | 0.261 | 0.971 | 0.331 | -0.258 | 0.765 |
| Average Stable | distressnov | -0.105 | 0.241 | -0.435 | 0.663 | -0.577 | 0.367 |
| Average Stable | struc_parenting | -0.227 | 0.215 | -1.058 | 0.29 | -0.649 | 0.194 |
| Average Stable | psychopathology | 0.488 | 0.383 | 1.274 | 0.203 | -0.263 | 1.239 |
| Average Stable | struc_parenting:psychopathology | -0.805 | 0.329 | -2.443 | 0.015 | -1.451 | -0.159 |
| Higher Stable | (Intercept) | 0.931 | 0.349 | 2.669 | 0.008 | 0.247 | 1.614 |
| Higher Stable | openness | 0.004 | 0.247 | 0.015 | 0.988 | -0.48 | 0.488 |
| Higher Stable | prenatal_drug | 0.306 | 0.45 | 0.679 | 0.497 | -0.576 | 1.188 |
| Higher Stable | distresslim | 1.039 | 0.284 | 3.656 | 0 | 0.482 | 1.595 |
| Higher Stable | distressnov | -0.179 | 0.262 | -0.684 | 0.494 | -0.692 | 0.334 |
| Higher Stable | struc_parenting | -0.143 | 0.233 | -0.612 | 0.541 | -0.599 | 0.314 |
| Higher Stable | psychopathology | 0.25 | 0.41 | 0.611 | 0.541 | -0.553 | 1.053 |
| Higher Stable | struc_parenting:psychopathology | -0.93 | 0.365 | -2.545 | 0.011 | -1.646 | -0.214 |
| High Increasing | (Intercept) | 0.35 | 0.387 | 0.903 | 0.367 | -0.41 | 1.109 |
| High Increasing | openness | -0.098 | 0.295 | -0.334 | 0.738 | -0.677 | 0.48 |
| High Increasing | prenatal_drug | -0.481 | 0.544 | -0.884 | 0.377 | -1.547 | 0.585 |
| High Increasing | distresslim | 0.89 | 0.338 | 2.635 | 0.008 | 0.228 | 1.553 |
| High Increasing | distressnov | -0.15 | 0.314 | -0.476 | 0.634 | -0.765 | 0.466 |
| High Increasing | struc_parenting | -0.421 | 0.274 | -1.539 | 0.124 | -0.957 | 0.115 |
| High Increasing | psychopathology | 0.633 | 0.451 | 1.405 | 0.16 | -0.25 | 1.517 |
| High Increasing | struc_parenting:psychopathology | -0.981 | 0.413 | -2.376 | 0.017 | -1.79 | -0.172 |

Note. distresslim = Distress to Limitation. distressnov = Distress to Novelty. struc_parenting = Structured Parenting. psychopathology = Biological Father Psychopathology.
